# A Rare Case of Cyclotorsion Due to Medial Rectus Displacement Following Orbital Trauma

**DOI:** 10.22599/bioj.165

**Published:** 2021-04-15

**Authors:** Elspeth Green, Hannah Harwood, Shveta Bansal

**Affiliations:** 1 Manchester Royal Eye Hospital, GB; 2 Royal Preston Hospital, GB

**Keywords:** Excyclotorsion, orbital fracture, medial rectus, inferior rectus

## Abstract

We describe a rare case of cyclotorsion likely secondary to medial rectus and inferior rectus pathology in a patient with orbital trauma. Sequential orthoptic measurements including Hess charts are presented alongside relevant sections of the orbital CT scans over the course of the patient’s treatment. Following the insertion of a plate to repair an orbital floor fracture, the patient developed cyclotorsion. A combined approach of sequential orthoptic assessment and imaging revealed the likely underlying mechanism. Inferior rectus mechanical restriction combined with displacement of the medial rectus pulley appear to be the likely culprits. Once the orbital plate was exchanged for a smaller sized plate the patient’s symptoms and clinical features resolved. Although orbital plate malpositioning is not an uncommon event, medial rectus deviation as a cause of cyclotorsion has not previously been described. We discuss the alternative differentials for patients with similar orthoptic findings and how they were excluded.

## Case

A 47-year-old patient presented to the hospital following an alleged assault involving multiple kicks to his face. He was found to have considerable ecchymosis around the right orbit, a right eye subconjunctival haemorrhage, and paraesthesia of the right side of his face. Visual acuity was better than 0.0 LogMAR in both eyes.

**Figure 1:**
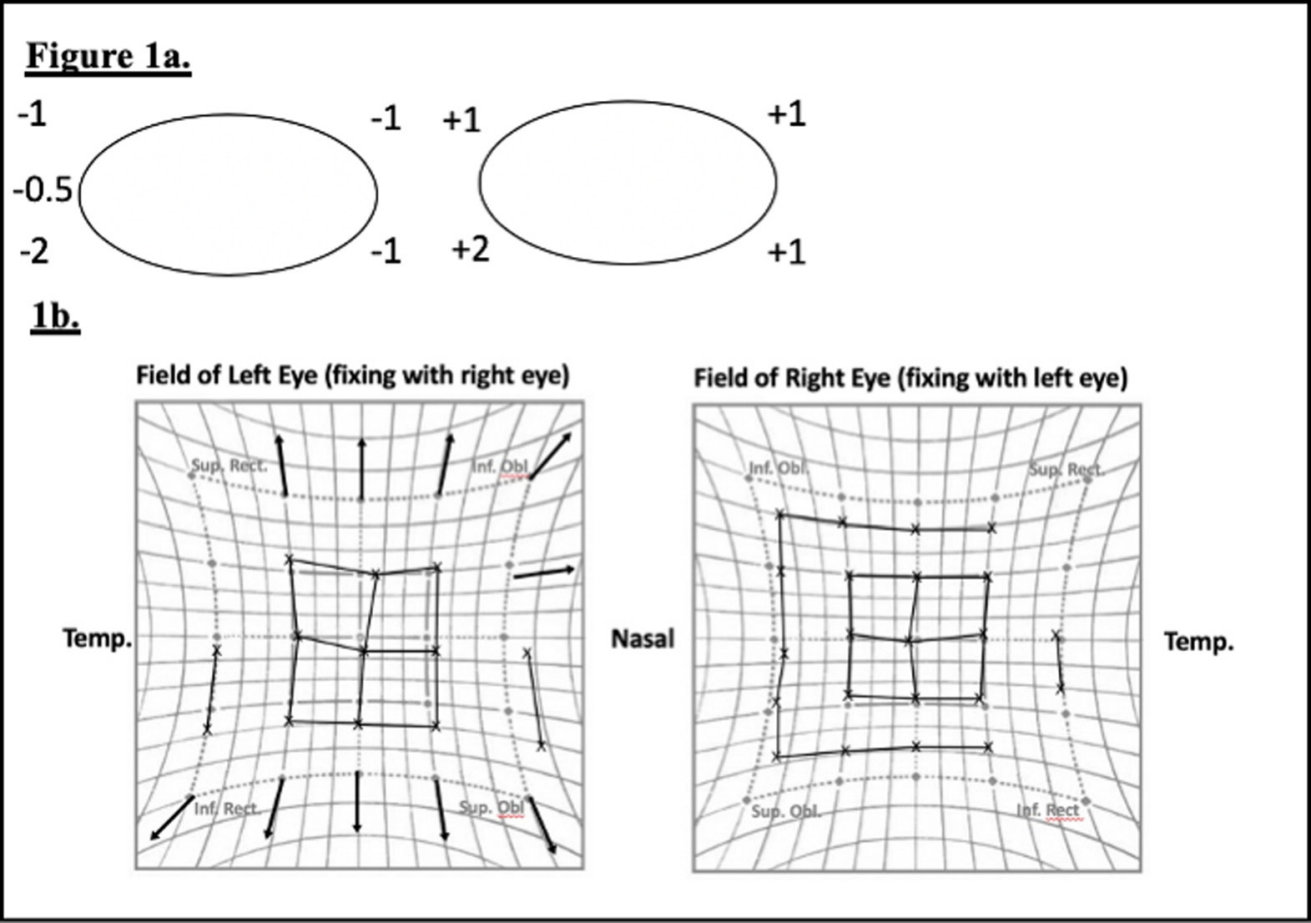
Preoperative clinical findings following initial injury. **1a.** Ocular motility, **1b.** Hess chart shows compression in the right eye.

**Figure 2:**
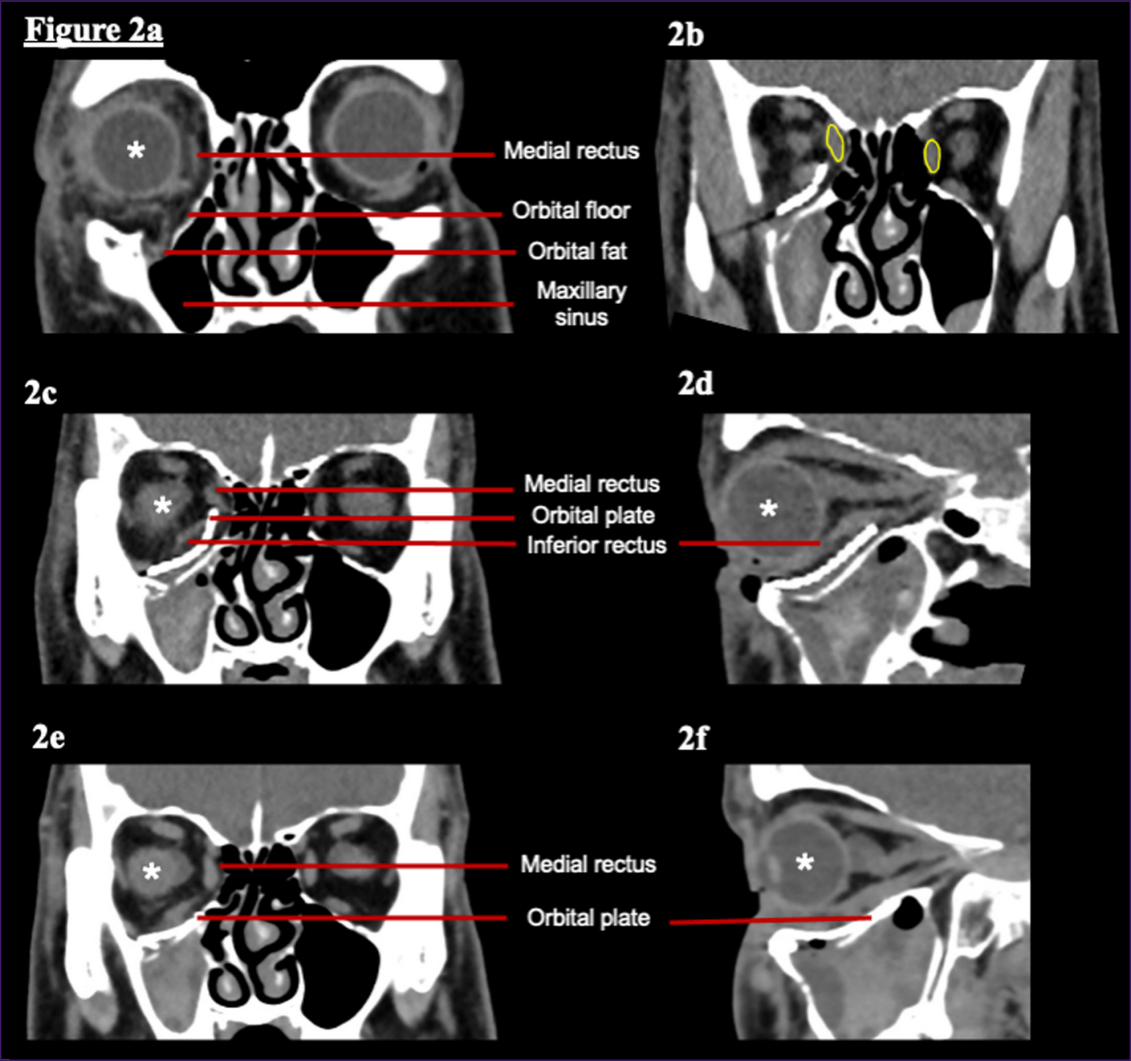
CT scan of orbit using soft tissue windows. **2a** Orbital floor fracture following initial injury. **2b.** Coronal section following orbital floor repair. The bodies of the left and right medial recti have been highlighted to demonstrate their asymmetric positioning. The right medial rectus is clearly elevated in comparison to the contralateral side. **2c.** Coronal section of the orbit shows the medial rectus tendon in close proximity to and distorted by the orbital plate. Anatomical structures are labelled, * indicates the globe. **2d.** Sagittal section shows posterior extension of orbital plate. **2e.** Coronal section following orbital plate exchange shows right medial rectus now symmetrical with left. **2f.** Sagittal section following orbital plate exchange shows less posterior extension of orbital plate.

At his initial assessment he reported mild horizontal and vertical diplopia in upgaze and downgaze only. His examination demonstrated generalised mild restriction in all positions of gaze in the right eye, maximal in upgaze. In primary position only an exophoria was present. This corresponded with the Hess chart findings which consisted of a compressed pattern in the right eye and an expanded pattern in the left eye (***Figure 1***). On the basis of these findings there was concern about muscle or orbital fat entrapment. A CT performed at this time demonstrated a right orbital floor fracture with orbital fat herniating into the maxillary sinus (***Figure 2***). An orbital floor reconstruction was performed 10 days later without complication. After the orbital plate was inserted a forced duction test confirmed the globe was freely mobile.

**Figure 3:**
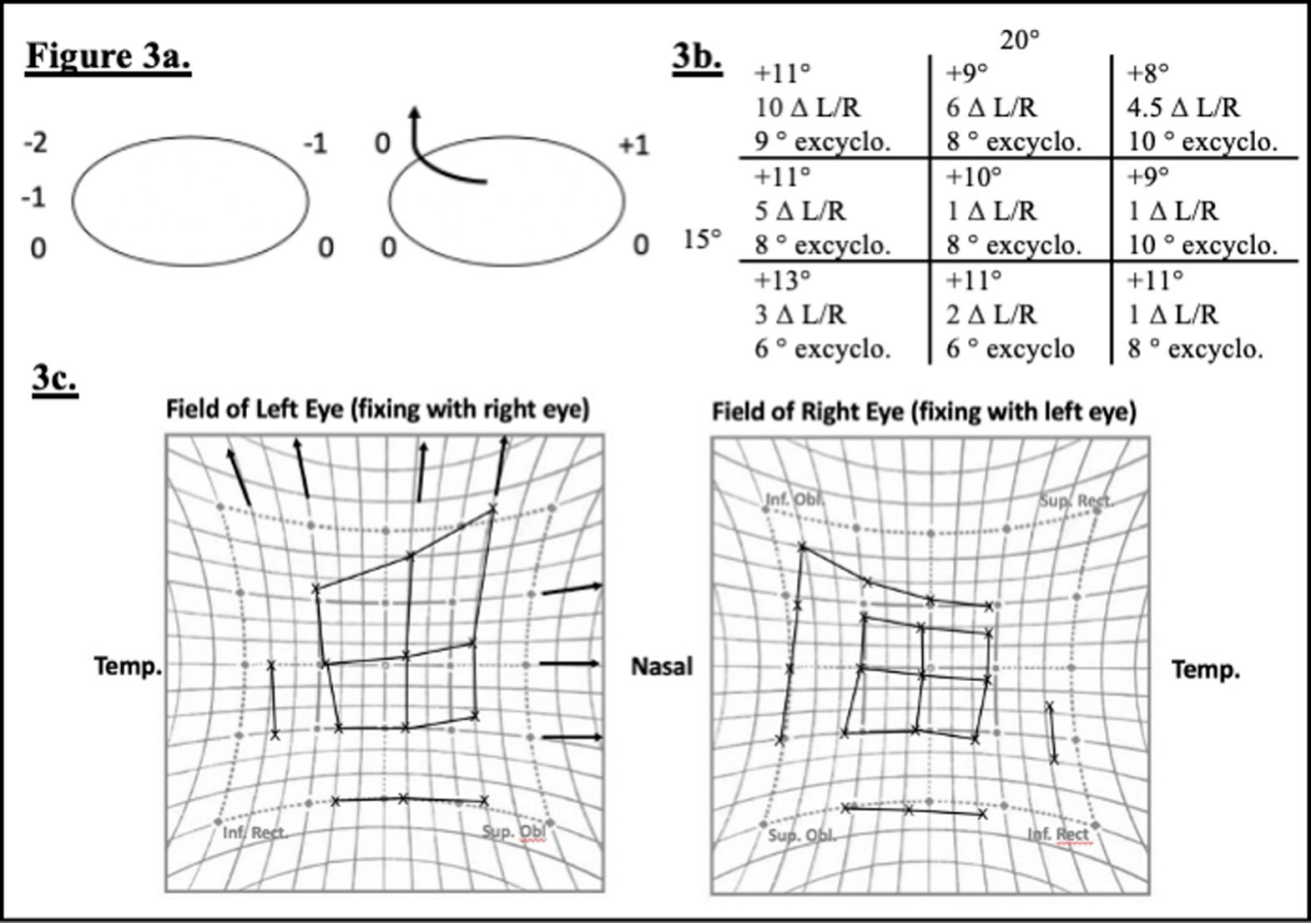
Postoperative clinical findings following orbital floor repair. **3a.** Ocular motility, **3b.** Synoptophore performed postoperatively with left eye fixating. Esotropia, excyclotorsion and mild left hypertropia noted in primary position. Left hypertropia maximal in dextroelevation. Esotropia was present in all positions of gaze and maximal in dextrodepression. Excyclotorsion was maximal in levoversion. **3c.** Hess chart showing a compressed appearance in the right eye. Note restriction of upgaze in the right eye with associated overaction of the left superior rectus and underaction of right inferior oblique, medial rectus and inferior oblique.

Immediately after surgery the patient complained of significantly increased vertical diplopia with new symptoms of torsion in all positions of gaze. He was found to have a right hypotropia and excyclotorsion maximal on levoversion of up to 10 degrees on synoptophore (***Figure 3***). There was also some limitation in upgaze that appeared to be due to mechanical restriction, particularly in dextroelevation.

A CT was performed, which showed changes in the position of medial rectus and inferior rectus following orbital plate insertion (***Figure 2***). The orbital plate elevated the right medial rectus and distorted the natural course of the muscle. When compared to the contralateral medial rectus, it was clear that the right medial rectus was elevated. ***Figure 2c*** demonstrates the medial rectus tendon appearing to ride on the border of the orbital plate. The inferior rectus is also seen in close apposition to the orbital plate. Based on imaging alone, entrapment or scarring between the muscle and plate could not be excluded. The superior rectus, lateral rectus, superior oblique and inferior oblique showed no abnormality on CT imaging.

Based on these clinical findings, combined with the CT, a diagnosis of medial rectus pulley distortion and possible inferior rectus mechanical restriction was made. The patient was offered a revision of the orbital plate to attempt to resolve the double vision. He elected to go ahead and 6 weeks after the injury the original plate was replaced with a smaller plate that extended less posteriorly and medially. No additional scarring or entrapment was found during the operation. After the plate was inserted the globe was freely mobile on forced duction testing.

**Figure 4:**
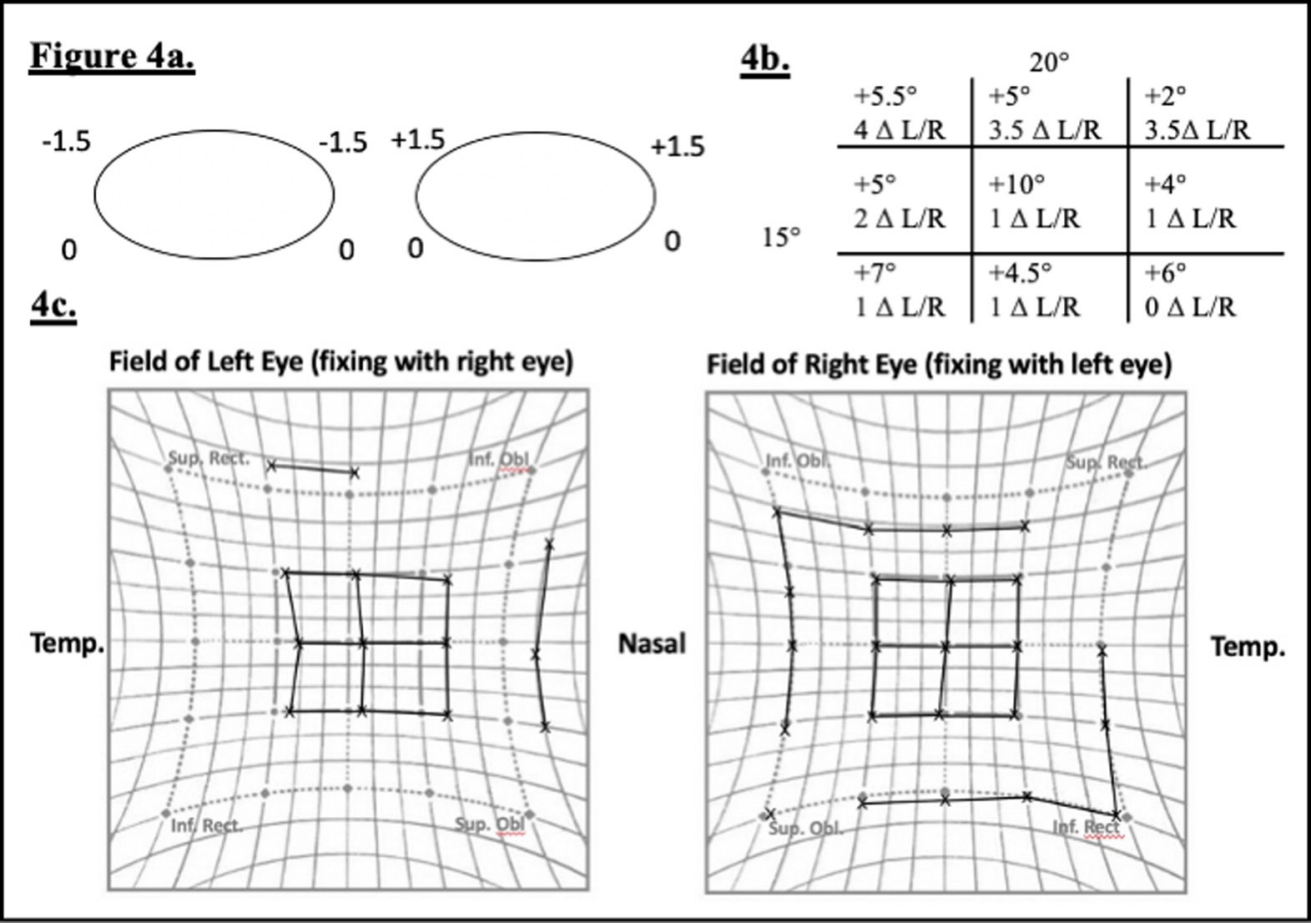
Postoperative clinical findings following orbital plate exchange. **4a.** Ocular motility, **4b.** Synoptophore, left eye fixation, no torsion in any position of gaze, **4c.** Hess chart.

Following his second operation the patient reported a total resolution of his symptoms. Examination revealed return of normal ocular motility and no significant torsion (***Figure 4***). The repeated CT scan showed return of the medial rectus to a normal position (***Figure 2***). He was able to resume all of his premorbid daily activities without any orthoptic aids.

## Discussion

This case demonstrates torsional diplopia, possibly contributed to by multiple sources. The underlying cause may have been mechanical restriction of the inferior rectus and associated overaction of the left inferior oblique. Alternatively, displacement of the medial rectus pulley due to the posterior positioning of the plate may have led the muscle to have a cyclotorting action on the globe. Extraocular muscle pulleys are flexible, supportive connective tissues that shift during contraction and relaxation to guide the path of action of the muscles (Miller 2007). When a muscle pulley is distorted, the muscle’s field of action will change (Clark & Demer 2002). It is likely a combination of the two mechanisms played a part.

To reach this conclusion, we examine the differential diagnosis of excyclotorsion following orbital fracture and exclude the different alternative diagnoses systematically. The underlying causes can be subdivided into neurological and mechanical causes. These include superior oblique or superior rectus palsy, or mechanical restriction of the extraocular muscles due to muscle entrapment, or postoperative scarring. Additionally, the vector of muscle pulley action can be altered by scar tissue or changes of the orbital structure.

Damage to the trochlea nerve causing a superior oblique palsy would be expected to result in excyclotorsion. However, it would also be associated with underaction of the muscle when the eye is adducted and depressed, whereas this patient had overaction of the right eye when looking ‘down and in’. Damage to the right oculomotor nerve and in particular the superior division could result in a superior rectus palsy leading to loss of intorsion in adduction. However, this patient had greater excyclotorsion in abduction, making this diagnosis unlikely.

One differential includes inferior oblique mechanical restriction or entrapment, which has previously been reported to result in extorsional diplopia (Kim, Kim & Kim 2009; Kakizaki et al. 2005). However, while the preoperative measurements show a degree of under action, mechanical restriction is not shown on the Hess chart, and imaging did not find any abnormality of the inferior oblique muscle. Underaction of the inferior oblique, as shown on the Hess chart has instead been associated with intorsional diplopia (Takahashi & Kakizaki 2019).

A number of case reports describe iatrogenic Brown syndrome following superior and medial orbital wall repairs (Lee et al. 2009; Seo et al. 2010; Lauer, Sauer & Pak 1998; Hwang & Lim 2005). In this case, Brown syndrome is not suspected, as there is no increase in excyclotorsion on adduction and the superior orbit and trochlea were not affected by the original injury or surgery.

Right inferior rectus entrapment with contralateral inferior oblique overaction would be expected to produce a similar Hess chart to that shown in ***Figure 3***. It would explain the compressed appearance of the right Hess chart, combined with left hypertropia maximal in dextroelevation. However, as the inferior rectus has a tertiary action of excyclotorsion, underaction of the inferior rectus alone would be expected to cause mild incyclotorsion.

Cyclotorsion may be attributable to the visible elevation of the medial rectus pulley posteriorly, seen on CT image in ***Figure 4a*** and ***4c***. The effect of this distorted pully would be to induce a right hypotropia and excyclotorsion. This was seen in all positions of gaze, but most marked in levoversion. This theory is supported by the resolution of symptoms following adjustment of the position of the orbital floor plate allowing the medial rectus to resume its normal path shown in ***Figure 4d*** and ***4e***.

No case reports have previously described distortion of the medial rectus pulley with associated torsion. A literature search performed using the keywords “medial rectus” and “cyclotorsion” revealed no studies that described cyclotorsion due to medial rectus pathology or surgery. We found several reports of medial rectus entrapment either due to orbital fractures or postoperative complications. These cases report entrapment resulting in adduction and abduction restriction, sometimes associated with globe retraction (Ela-Dalman, Velez & Rosenbaum 2006; Lee et al. 2009). In the case we have described above the imaging and clinical findings were not suggestive of medial rectus entrapment.

Orbital plate insertion is known to potentially result in complications, most frequently diplopia, enophthalmos and ectropion. Diplopia may occur following orbital reconstruction as a result of paresis of the muscle due to the original injury, scarring of orbital tissues or malposition of the prosthesis resulting in muscle entrapment (Zimmerer et al. 2018; Mauiello JA 1990). A recent retrospective study of 71 patients undergoing orbital repair with a titanium mesh found that 23% of implants were poorly positioned, and 17% required revision (Schlittler F et al. 2018). In a separate study investigating the indication for secondary orbital repair, extraocular muscle entrapment was the indication in 15% of patients (Kim JS et al 2016). In patients that have previously undergone orbital floor repair and secondary repair is required, the inferior rectus is the most likely muscle to be involved (Mauiello JA 1990). Fortunately, ocular motility restriction may not always result in symptoms; out of a cohort of 53 patients requiring orbital floor fracture repair, although 9.4% developed a new restriction after surgery, and a total of 22.6% of patients experienced diplopia postoperatively, the overall number of patients aware of diplopia was 13.2%. This highlights the limitation of only assessing surgical outcomes by subjective reports of diplopia or confrontation with a finger or pencil in primary position. A formal orthoptic assessment will enable clinicians to examine a full range of eye movement and identify the underlying cause of any symptoms.

This case illustrates the value of a multi-disciplinary approach to the patient’s management. Correlating the orthoptic findings with the CT findings allowed a surgical plan to be made and executed successfully. Ocular motility examination allowed the exclusion of possible entrapment or mechanical restriction of the inferior oblique. It also allowed clinicians to exclude a superior oblique underaction as a cause of the diplopia. The CT excluded abnormality of the superior rectus, as well as successfully showing the medial rectus deviation.

## Conclusion

We report the first case of medial rectus deviation due to a posterior and medially positioned orbital plate causing excyclotorsion, likely in combination with inferior rectus restriction. This case illustrates the importance of a combined orthoptic assessment and CT imaging in order to reach a diagnosis and formulate a successful treatment plan. An important learning point is that the function of a muscle can be significantly changed by displacement of its pulley. These changes cannot be identified by an orthoptic report alone, although unexplained findings such as excyclotorsion in this case should raise clinical suspicion.
